# Function of alveolar macrophages in lung cancer microenvironment

**DOI:** 10.1186/s41232-024-00335-4

**Published:** 2024-05-08

**Authors:** Takahiro Matsui, Seiji Taniguchi, Masaru Ishii

**Affiliations:** 1 Department of Immunology and Cell Biology, Osaka University Graduate School of Medicine, Suita, Osaka Japan; 2 Department of Pathology, Osaka University Graduate School of Medicine, Suita, Osaka Japan; 3 Department of Thoracic Surgery, Osaka University Graduate School of Medicine, Suita, Osaka Japan; 4 Department of Thoracic Surgery, Osaka Habikino Medical Center, Habikino, Osaka Japan

**Keywords:** Alveolar macrophage, Tissue-resident macrophage, Lung cancer, Cancer microenvironment, *INHBA*, Activin A

## Abstract

**Background:**

Cancer tissues contain a wide variety of immune cells that play critical roles in suppressing or promoting tumor progression. Macrophages are one of the most predominant populations in the tumor microenvironment and are composed of two classes: infiltrating macrophages from the bone marrow and tissue-resident macrophages (TRMs). This review aimed to outline the function of TRMs in the tumor microenvironment, focusing on lung cancer.

**Review:**

Although the functions of infiltrating macrophages and tumor-associated macrophages have been intensively analyzed, a comprehensive understanding of TRM function in cancer is relatively insufficient because it differs depending on the tissue and organ. Alveolar macrophages (AMs), one of the most important TRMs in the lungs, are replenished in situ, independent of hematopoietic stem cells in the bone marrow, and are abundant in lung cancer tissue. Recently, we reported that AMs support cancer cell proliferation and contribute to unfavorable outcomes.

**Conclusion:**

In this review, we introduce the functions of AMs in lung cancer and their underlying molecular mechanisms. A thorough understanding of the functions of AMs in lung cancer will lead to improved treatment outcomes.

## Background

It goes without saying that the cancer cells play the most important role in the tissue of malignant neoplasms. However, it is also widely known that neoplastic tissues contain a wide variety of non-neoplastic cells in addition to tumor cells, constituting unique tumor microenvironments. Many types of immune cells play critical roles in the suppression or promotion of tumor progression. For example, CD8^+^ T cells [1], natural killer cells [2], and myeloid cells, such as neutrophils [3] have a major influence on cancer progression through their interactions with cancer cells. In addition to direct cell-to-cell interactions, the effects of cytokines produced by tumor cells are important for the recruitment of tumor tissue-specific immune cells.

Macrophages, originally known to play a role in maintaining homeostasis by sensing immune signals, performing phagocytosis, and orchestrating subsequent responses, are also important tumor tissue-specific immune cells. They are the most abundant immune cell population in the tumor microenvironment, representing approximately 50% of hematopoietic cells [4]. Immunological studies have revealed that macrophages broadly comprise two classes: infiltrating macrophages and tissue-resident macrophages (TRMs) [5]. Previous reports have revealed that Ly6c^+^ monocytes from the bone marrow are the source of infiltrating macrophages found in pathological settings such as atherosclerosis and metabolic diseases [5]. In cancer tissues, most macrophages differentiate from bone marrow-derived monocytes and are mobilized to the cancer microenvironment via systemic circulation. They are called tumor-associated macrophages (TAMs) and are considered to support cancer cell growth and metastasis, and mediate immunosuppressive effects on the adaptive immune cells of the tumor microenvironment [4]. In contrast, TRMs originate from hematopoietic progenitors in the yolk sac during embryonic development. TRMs can self-maintain independently from hematopoietic stem cells (HSCs) in the bone marrow and exhibit several microenvironment-specific phenotypes and functions in various tissues in the body [5, 6]. TRMs include microglia in the central nervous system, Kupffer cells in the liver, and Langerhans cells in the skin; most tissues in the body contain TRM populations [7]. These findings suggest that cancer tissues contain TRMs in addition to infiltrating macrophages or TAMs. However, although previous studies have reported the interactions between some TRMs and malignant tumors such as glioma, hepatocellular carcinoma, and metastatic cancer, the exact functions and roles of TRMs in cancer tissues are not fully understood [8–10].

Lung cancer is a malignant neoplasm that causes the largest number of deaths worldwide and is one of the most difficult diseases to cure [11]. Although therapeutic advances in immune checkpoint inhibitors have improved clinical outcomes to some extent [12], the results remain unsatisfactory. It is necessary to analyze each component of the lung cancer tissue in detail to fundamentally solve these problems. In this review, we focus on alveolar macrophages (AMs), major TRMs in the lungs, and outline their role and function in the lung cancer microenvironment.

## Main text

### Functions of AMs in non-tumor tissues

Owing to respiration, pulmonary macrophages play an important role in defending against foreign substances and pathogens to ensure lung homeostasis. There are two main components of TRMs in the lungs: AMs and interstitial macrophages. AMs are the predominant population, accounting for 90–95% of pulmonary resident immune cells [13]. As they reside on the luminal side of alveolar spaces [14], AMs are spatially distinguished from other lung macrophages located beneath the airway epithelium or around blood vessels. In addition, AMs exhibit unique developmental characteristics. Initial colonization of the airways with AMs is detectable in the first few days after birth and is wholly dependent on fetal monocytes [15, 16]. Many experimental models, such as parabiosis [16] and transplantation [17] have revealed that AMs have a marked capacity for self-renewal [15]. Therefore, AMs do not rely on blood monocytes from the bone marrow for their renewal and are replenished in situ throughout life, at least under steady-state conditions [5, 7, 18]. In addition, a recent study regarding the lineage analysis of macrophages in lung cancer tissues, using a mouse model with tamoxifen-inducible adult HSC-specific *CreER* transgenic mice, reported that tumor-associated AMs in mice and their homologous clusters in humans also arise from the TRM lineage, independent of adult HSCs [19].

AMs, together with the alveolar epithelium, play a central role in protecting against the outside environment and are closely linked to the alveolar epithelium. AMs are conjunct to alveolar epithelial cells and receive granulocyte–macrophage colony-stimulating factor (GM-CSF) from epithelial cells [8, 15]. GM-CSF, encoded by the colony-stimulating factor 2 (*Csf2*) gene, is necessary for the differentiation of fetal monocytes into AMs, and macrophages found in the airways of *Csf2* knockout (KO) mice cannot fully differentiate into AMs [16]. Another important factor for AM function is peroxisome proliferator-activated receptor‑γ (PPAR-γ), famous as a master regulator of adipocyte differentiation [20] and lipid metabolism. GM-CSF as well as transforming growth factor-β (TGF-β) signaling pathways activate this transcription factor to facilitate the differentiation and survival of AMs [21]. While PPAR-γ is expressed by both human and mouse AMs [15], its expression is lost in *Csf2*-KO mice [16]. A series of AM dysfunctions based on these mechanisms result in the accumulation of pulmonary surfactant lipids and proteins produced by type 2 alveolar epithelial cells in the airways, as the remaining macrophages are unable to catabolize them [22]. Clinically, AM dysfunction is known as pulmonary alveolar proteinosis and is mainly due to non-functional signaling by autoantibodies against GM-CSF [22].

In mice, AMs are characterized by the expression of CD45^+^, autofluorescence^+^, CD11c^+^, CD11b^ − ^, F4/80^+^, and sialic acid-binding immunoglobulin-like lectin F^+^ [23], while in humans, they are identified by autofluorescence^+^, HLA-DR^+^, CD43^+^, CD88^+^, CD169^+^, CD206^+^, CD11c^+^, CD141^+^, CD64^+^, CD71^+^, and CD163^+^ [14, 24]. Autofluorescence detection is very effective for isolation by flow cytometry [23, 25].

### Functions of AMs in lung cancer tissue

Lung cancer is a malignant tumor of the alveolar epithelium that constitutes the alveolar space. Similar to the AM, the alveolar epithelium is constantly exposed to many external stimuli due to constant respiration, which is thought to be one of the major causes of carcinogenesis and promotion [26]. Histologically, it seems obvious that AMs are the first immune cells to contact cancer cells soon after development. However, despite the histological evidence, little research has been conducted on the interactions between lung cancer cells and AMs. One of the main reasons why AM analysis is difficult is that it is difficult to analyze TRMs ex vivo. It is possible to collect AMs from bronchoalveolar lavage fluid to obtain primary cells. For long-term in vitro analysis, AM cell lines are available [27]. In an analysis using cell lines derived from the C57BL/6 mouse strain, the addition of AM cell culture supernatants (AMJ2-C11) promoted the growth of lung cancer cells (Lewis lung carcinoma) [25], suggesting that AMs could influence the proliferation of lung cancer cells via the secretion of soluble factors.

In addition to conventional cell biological analysis, it has recently become possible to obtain precise information through transcriptome analysis, not only from in vivo experimental models but also from clinical human samples. Recent studies involving patients with non-small cell lung cancer (NSCLC) and a mouse model, in which tail vein injections of *Kras*^*G12D*^-transduced and *p53*-deficient lung epithelial cells were used, have highlighted the significant role of tissue-resident AMs in driving lung tumorigenesis [19]. It is important to note that AM benefits lung cancer cells, similar to the in vitro experiments mentioned previously. Histologically, tumor cells were localized close to the AMs after tumor seeding in a mouse model with tail vein infusion of cancer cells. Moreover, a similar redistribution of AMs was observed in a genetically engineered mouse lung cancer model [19]. Such accumulation of AMs close to tumor cells was observed early during tumor formation. In contrast, infiltrating macrophages or TAMs dominated in advanced tumor lesions, suggesting that the effects of AMs on lung cancers might be particularly pronounced in early lesions. In addition, coculture of lung cancer spheroids with macrophages indicated that AMs accumulate early during tumor formation to promote epithelial-mesenchymal transition (EMT), alter epithelial cell junctions accompanied by upregulation of the transcription factor *TWIST1*, and reduce expression of E-cadherin. EMT drives the acquisition of an invasive phenotype and AMs promote tumor cell invasiveness during the initial stages of tumor progression [19]. Moreover, another protumor effect via interactions with immune cells has been reported. Depletion of AMs using a deleter mouse line in which the diphtheria toxin receptor is expressed under the control of the CD169 promoter reduces the number and alters the phenotype of regulatory T cells and promotes the accumulation of CD8^+^ T cells [19]. These results indicate that AMs induce potent regulatory T cell responses to protect tumor cells from being attacked by CD8^+^ T cells.

**Fig. 1:**
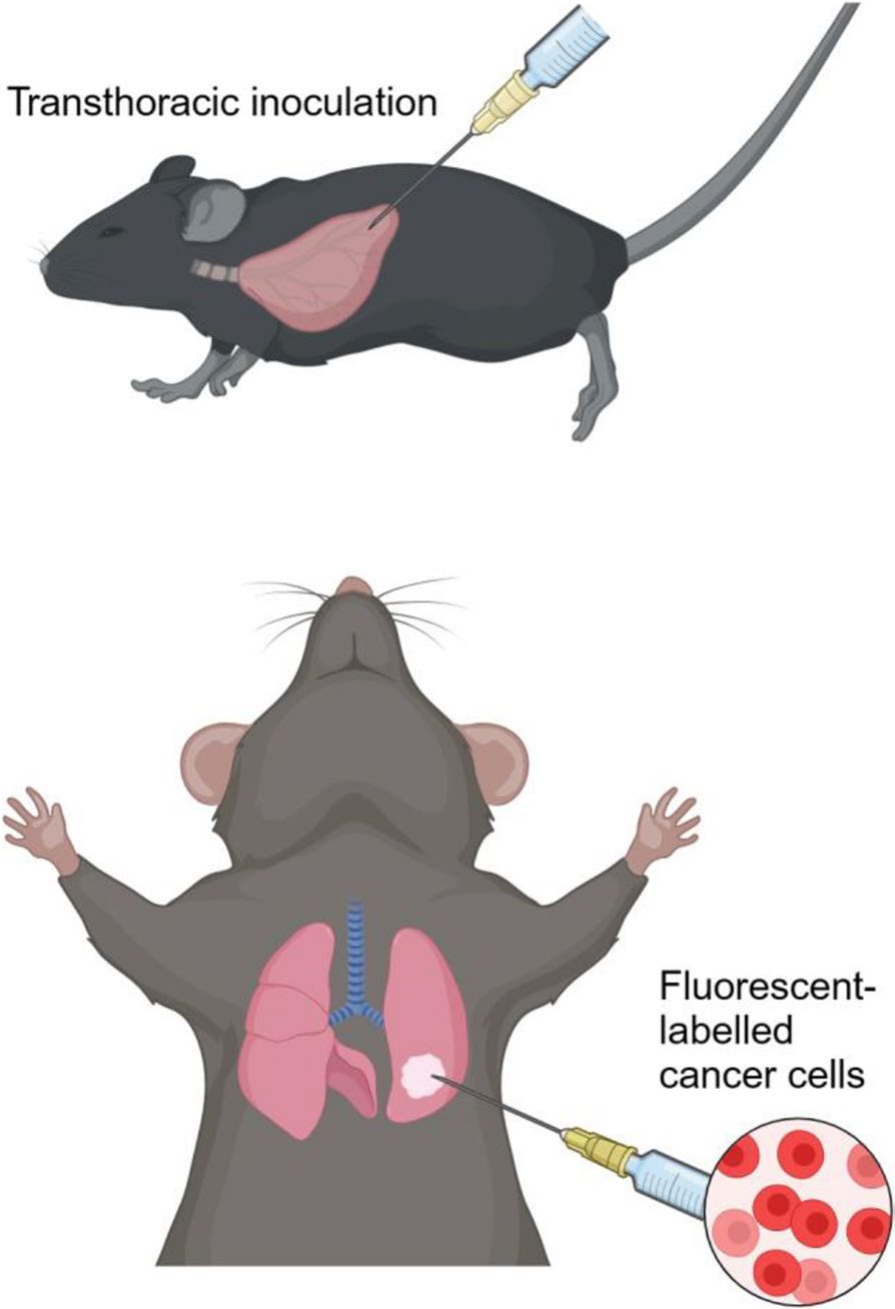
The schematic diagram of the orthotopic lung cancer model. Mice were anesthetized and placed in the right lateral decubitus position. A small skin incision to the left chest wall was made parallel to the ribs to inoculate cancer cells in the left lung

Casanova-Acebes et al. reported an undesirable function of AMs in lung cancer using both mouse models and clinical samples [19]. However, specific factors that trigger cancer cell transformation remain unclear. Moreover, the tail vein injection model is closer to a hematogenous metastasis model, and analysis using another experimental model is important. We employed a different experimental mouse model, an orthotopic lung cancer model, using tumor cell inoculation directly into the left lung (Fig. 1) [25]. Analysis using this lung cancer model in *Csf2*-KO mice also indicated that the tumor size was smaller in the absence of AMs. Similar results were obtained from the analyses of AM-depleted mice by intratracheal administration of clodronate liposomes, a reagent for macrophage-specific depletion [25, 28].

Furthermore, bulk RNA sequence analysis using this mouse model identified the inhibin subunit beta A (*INHBA*) gene to be specifically upregulated in AMs in the tumor microenvironment [25]. Overexpression of the *INHBA* gene, observed in the transcriptome analysis of early-stage human NSCLC samples as well [19], results in the production of activin A (ActA), the homodimer of the INHBA subunit. ActA is a member of the TGF-β superfamily and plays an important role in embryonic stem cell differentiation [29]. Previous reports on cancer biology have indicated that ActA also facilitates cancer progression [30] and promotes cell invasion in lung cancer via EMT [31]. Although these reports suggested an autocrine mechanism in tumor cells, a recent study indicated that ActA was significantly expressed in AMs of the tumor microenvironment compared to control AMs, other hematopoietic cells, and tumor cells using quantitative PCR analysis and enzyme-linked immunosorbent assay [25]. Recombinant ActA promotes lung cancer cell proliferation in vitro, and treatment with follistatin, an antagonist of ActA, significantly inhibits tumor formation in vivo. Similarly, tumor proliferation was suppressed in *INHBA*-conditional KO mice. These results indicate that ActA produced from AMs in the tumor microenvironment supports the proliferation of lung cancer cells in vivo [25]. Moreover, because we also confirmed that AM depletion led to metastasis inhibition to contralateral lung lobes in vivo, we concluded that ActA production by AMs may affect not only proliferation ability but also cancer stemness via EMT [31]. We further performed single-cell RNA sequence analysis, indicating that tumor-bearing conditions, such as damage-associated molecular pattern molecules, made some AMs constitute *INHBA*-expressing subclusters distinct from the ones in normal lungs [25]. Our analysis with mouse primary AM cells indicated that *INHBA*-expressing AMs in the tumor microenvironment showed reduced expression of a scavenger receptor (macrophage receptor with collagenous structure) compared to the normal counterpart [25]. Therefore, the *INHBA*-expressing AMs in lung cancer might have functional changes in phagocytosis compared to AMs in normal tissues. INHBA/ActA expression in AM is induced via Toll-like receptor 4, MyD88, and c-Jun N-terminal kinase signaling [25].

**Fig. 2:**
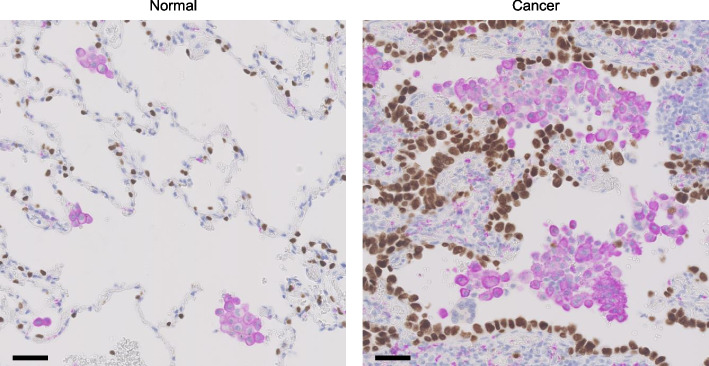
Immunohistochemical staining of the normal lung area (left) and lung cancer area (right) from patients with lung cancer. Purple cells indicate CD163^+^ macrophages and brown nuclei indicate alveolar epithelial cells or carcinoma cells positive for thyroid transcription factor-1. Scale bars; 50 μm

**Fig. 3:**
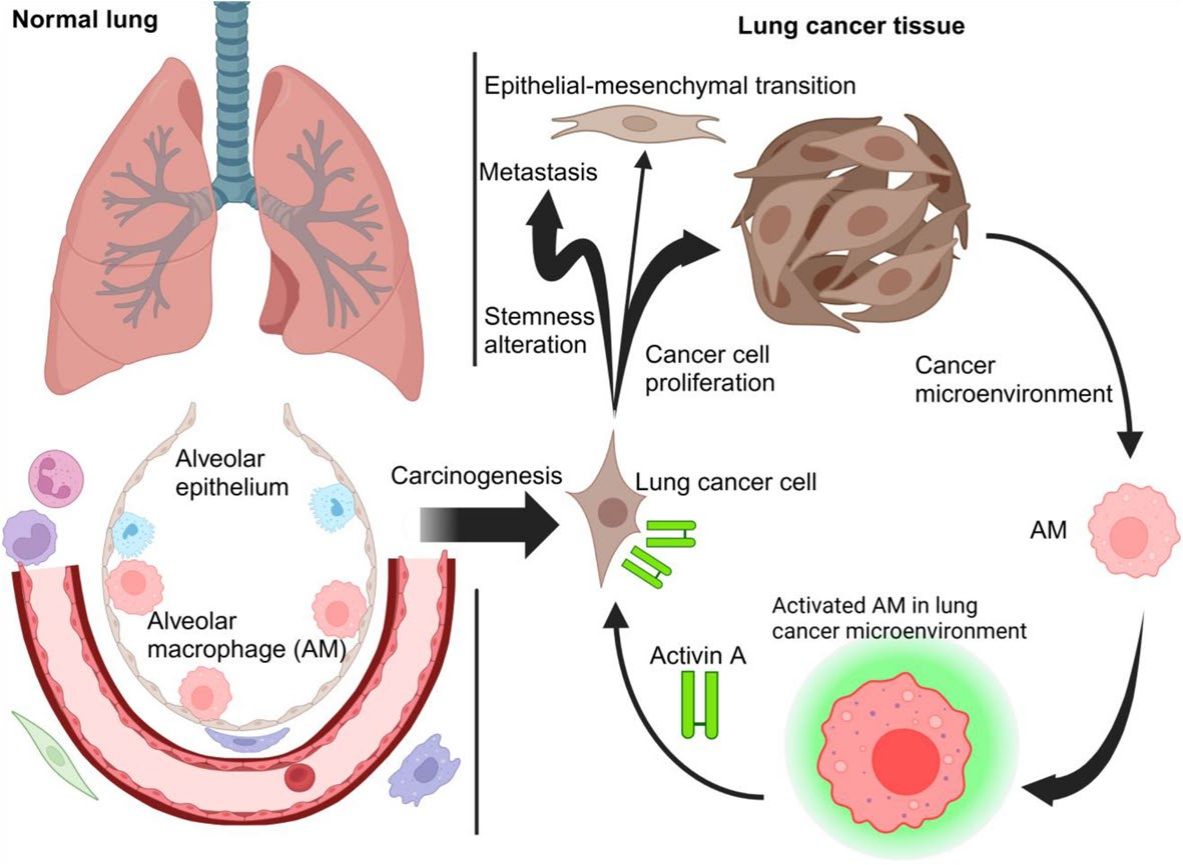
The schematic diagram of the activin A-producing AM subtype in the lung cancer microenvironment

We also confirmed in human tissue analysis that AMs in lung cancer are increased in both number and proportion compared to normal conditions (Fig. 2). The mechanism of AM proliferation in lung cancer seems to be similar to that in normal lungs because a recent study with lung adenocarcinoma mouse models indicated that GM-CSF secretion from the epithelium induced AM proliferation driven by GM-CSF-PPAR-γ signaling [32]. We further verified that *INHBA*-high AMs were present in human lung cancer tissues by immunohistochemical staining and single-cell transcriptomes from human lung samples using a public dataset. Moreover, another research with human lung cancer tissue samples also indicated that patients with high AMs in the peri-tumoral lung field showed significantly shorter recurrence-free and shorter overall survival than those with low AMs [33]. These analyses indicated that AMs exhibit a specific phenotype in the tumor microenvironment and play an important role in the progression of lung cancer (Fig. 3).

## Conclusions

Most previous reports have indicated that AMs promote tumor progression in a lung cancer microenvironment. These findings are similar to those in a report about other lung TRMs (interstitial macrophages) [34]. Furthermore, various mouse models of lung cancer and transcriptome analyses have revealed their molecular background. It is also noteworthy that the interaction between AMs and tumor cells is observed in the early stages of lung cancer. Suppression of AM function may facilitate treatment at an early stage and effectively inhibit lung cancer progression. Therefore, the inhibition of ActA signaling may be a promising option. Furthermore, it may be possible to develop an early cancer diagnosis by focusing on AMs. As cancers in the early stage often show few morphological changes, it is important to combine as much information as possible including non-neoplastic cells, to detect early cancer [35]. It has already been reported that serum levels of ActA and its inhibitor follistatin increased in lung cancer patients in a stage-dependent manner [36, 37]. This suggests that ActA signaling could be a promising biomarker for early detection of lung cancer. Furthermore, ActA may also be involved in the mechanism mediated by components other than cancer cells. A recent study indicated that ActA mediated cross-talk between cancer cells and cancer-associated fibroblasts in the lung metastatic niche and enhanced fibrosis and metastasis [38]. Alternatively, there also exists a report of in vitro experiments with a human cell line indicating that ActA promoted apoptosis of lung cancer cells [39]. Therefore, a more detailed analysis of ActA’s mechanism of action is needed. We believe that a detailed understanding of all components of cancer tissue, including AMs, will contribute to a complete cure for lung cancer in the future.

## Data Availability

Not applicable.

## Abbreviations

ActA: Activin A
AM: Alveolar macrophage
*Csf2*: Colony stimulating factor 2
EMT: Epithelial–mesenchymal transition
GM-CSF: Granulocyte macrophage colony-stimulating factor
HSC: Hematopoietic stem cell
*INHBA*: Inhibin subunit beta A
KO: Knockout
NSCLC: Non-small cell lung cancer
PPAR-γ: Peroxisome proliferator activated receptor gamma
TAM: Tumor-associated macrophage
TRM: Tissue-resident macrophage
TGF-β: Transforming growth factor-β
